# Associations of Changes in Skeletal Muscle Mass on Computed Tomography with Improvements of Muscle Strength and Physical Activity after Sleeve Gastrectomy: A Pilot Study

**DOI:** 10.1298/ptr.25-E10351

**Published:** 2025-09-02

**Authors:** Misuzu KUBA, Shinichiro UEDA

**Affiliations:** 1 Department of Rehabilitation Therapy, Ohama Dai-ichi Hospital, Japan; 2 Department of Clinical Research and Quality Management, Graduate School of Medicine, University of the Ryukyus, Japan

**Keywords:** Skeletal muscle density, Relative muscle strength, Physical activity, CT images, Sleeve gastrectomy

## Abstract

**Objective:**

Sleeve gastrectomy (SG) effectively reduces food intake and enhances health and longevity, although weight loss often involves reductions in body fat and skeletal muscle. Hence, we examined how changes in skeletal muscle density relate to muscle strength and physical activity before and after SG.

**Methods:**

This pilot observational study included 173 patients (mean age: 46 ± 11 years) who attended the hospital before and 1 year after undergoing SG between December 2018 and July 2022. Muscle cross-sectional area (cm^2^), skeletal muscle density, and fat cross-sectional area (cm^2^) at the 3rd lumbar vertebra were measured on computed tomography (CT) images. The psoas muscle index (PMI) was calculated as the cross-sectional area of both psoas muscles (cm^2^) divided by height squared (m^2^), and CT values for skeletal muscle density of the psoas and multifidus muscles, visceral fat area (VFA), and subcutaneous fat area (SFA) were obtained. Relative and absolute muscle strengths, sitting time, and total physical activity from the International Physical Activity Questionnaire were also assessed.

**Results:**

Although VFA, SFA, PMI, and absolute muscle strength decreased significantly after SG compared to those before SG (p <0.001), skeletal muscle density, relative muscle strength, and total physical activity increased significantly after SG, with a significant reduction in sitting time (p <0.001). There were significant positive correlations between changes in skeletal muscle density and changes in relative muscle strength and total physical activity.

**Conclusions:**

Improving skeletal muscle density with SG may be associated with increased relative muscle strength and improved physical activity.

## Introduction

Sleeve gastrectomy (SG) is effective in reducing food intake and improving obesity-related health problems^1,2)^, improving quality of life (QOL)^2)^, and increasing healthy longevity, among other benefits^3)^. However, weight loss after SG is mainly due to reduced body fat, including visceral fat area (VFA) and subcutaneous fat area (SFA)^4)^, as well as reduced skeletal muscle mass^5–7)^, resulting in reduced absolute muscle strength (AMS) and increased relative muscle strength (RMS)^5,6)^.

Methods used to quantitatively assess skeletal muscle mass include computed tomography (CT), magnetic resonance imaging (MRI), and dual-energy X-ray absorptiometry^8)^. When assessing skeletal muscle mass in terms of muscle cross-sectional area measured by these modalities, qualitative changes in noncontractile tissue, such as fat infiltration of skeletal muscle, may lead to an overestimation of skeletal muscle mass. CT (X-ray absorptiometry: skeletal muscle density) is considered a useful index for the accuracy and reproducibility of skeletal muscle measurements; therefore, it is important to perform both quantitative and qualitative assessments of skeletal muscle^8,9)^. In CT-based assessments, the psoas muscle index (PMI), measured as the cross-sectional area of the psoas muscle at the level of the 3rd lumbar vertebra, is a useful diagnostic standard for skeletal muscle mass in Asian adults^10)^. For qualitative assessment of skeletal muscle, the CT value calculated from the muscle cross-sectional area at the level of the 3rd lumbar vertebra, measured in Hounsfield units (HU) or similar units, reflects skeletal muscle steatosis and can be used as an index of fat accumulation^9)^. These assessments have standardized criteria for reporting muscle attenuation^11)^ and are therefore widely used in areas such as obesity^12)^, malignancy^13,14)^, and cardiovascular disease^15)^. They are associated with declining physical function^12,15–17)^ and life expectancy^13–16)^. Skeletal muscle steatosis is also associated with muscle atrophy, and in addition to quantitative changes in the form of a reduction in the muscle fiber cross-sectional area, qualitative changes, such as fatty infiltration within the skeletal muscle, mean that a larger proportion of the muscle area is occupied by noncontractile tissue^8,11,18)^, which are associated with reduced muscle strength, declining physical function^12,15–17)^, and decreased physical activity^19)^. Although decreases in body fat, skeletal muscle mass, and muscle strength associated with post-SG weight loss have been widely reported^5–7)^, the associations of myosteatosis and other qualitative changes in skeletal muscle on CT with muscle strength and physical activity level remain unclear.

The aim of this study was to examine changes in skeletal muscle mass and density, as measured by CT, as well as changes in muscle strength and physical activity levels before and after SG, to determine how skeletal muscle density, an indicator of skeletal muscle quality, is related to muscle strength and physical activity levels.

## Methods

### (1) Ethics statement

This study was approved by the University of the Ryukyus Ethics Committee for Life Sciences and Medical Research Involving Human Subjects (approval number: 23-2145-00-00-00). Since this study was based solely on existing medical records, the requirement for written and oral informed consent was waived. However, information about the study was made available to the study participants, who were guaranteed the opportunity to decline participation.

### (2) Study design and population

Of the 255 patients who underwent SG for morbid obesity at Ohama Dai-ichi Hospital between December 2018 and July 2022, 173 men and women who attended our hospital for 1 year after SG were included in this pilot observational study.

To understand patients’ pre- and postoperative diet and exercise regimens, a dietitian assessed their nutritional status through interviews focused on dietary content and eating habits before and after SG. The objectives of nutritional management were healthy weight loss and weight maintenance. To this end, patients’ protein intake, supplement intake status, snacking and drinking habits, and dietary balance were assessed. Additionally, their body composition, muscle strength, and physical activity were monitored under the guidance of a physical therapist before and after SG. Feedback was provided based on the results to promote patient motivation and mental health.

Data on comorbidities were collected from the patients’ medical records. Body measurements, blood biochemistry test results, skeletal muscle mass, skeletal muscle density, body fat measured on CT images, muscle strength assessments, and International Physical Activity Questionnaire (IPAQ) results from before and 1 year after SG were evaluated.

### 1) Body measurements

Height, weight, and body mass index (BMI) were measured with a body composition analyzer (TANITA-MC 250; TANITA, Tokyo, Japan) during an early morning, fasting outpatient appointment.

### 2) Blood biochemistry tests

The following items were measured during an early morning, fasting outpatient appointment: hemoglobin A1c (HbA1c), fasting plasma glucose (FPG), aspartate transaminase (AST), alanine transaminase (ALT), γ-glutamyl transferase (γ-GPT), high-density lipoprotein cholesterol (HDL-C), low-density lipoprotein cholesterol (LDL-C), serum triglycerides (TG), and homeostasis model assessment of insulin resistance (HOMA-IR).

### 3) Skeletal muscle mass, skeletal muscle density, and body fat

CT images were analyzed using the diagnostic imaging software Ziostation2 (Ziosoft, Tokyo, Japan). During this process, the skeletal muscle area (−29 to 150 HU) was measured. The VFA and SFA were calculated by a radiological technologist, and the PMI and CT values were calculated by a physical therapist. All measurement procedures were standardized and rehearsed prior to the study.

Skeletal muscle mass, skeletal muscle density, and body fat were measured on CT images in the transverse section at the level of the 3rd lumbar vertebra. On these cross-sectional images, both psoas major muscles were traced as regions of interest (ROIs), their cross-sectional areas (cm^2^) were each measured twice, and the mean value was calculated. The PMI was calculated as the cross-sectional area of both psoas major muscles (cm^2^) divided by the square of the height (m^2^), and this was used as an index of skeletal muscle mass^10)^. Skeletal muscle density was evaluated by measuring the mean CT values of ROIs in the psoas major and multifidus muscles twice each and calculating the mean CT value in HU^9–11)^. The higher the HU value, the less fat infiltration is present, and the better the quality of the skeletal muscle^9,11)^. The VFA and SFA were calculated from the amount of body fat^20)^.

### 4) Muscle strength assessment

Grip strength was measured twice for each hand using a digital grip strength tester (TKK 5101 Grip-D; Takei, Tokyo, Japan), with the highest value of the 2 used as the maximum muscle strength value. Maximum grip strength was defined as AMS (kgf), and RMS (kgf/kg) was calculated by dividing absolute grip strength (kgf) by body weight (kg).

### 5) Physical activity level assessment

Physical activity levels were assessed using the Japanese version of the IPAQ. Total physical activity (TPA) (metabolic equivalent of task [MET]-hours/week), which combines the mean weekly levels of high-, moderate-, and low-intensity physical activity with walking frequency and duration, was calculated. TPA and sitting time (ST) (hours/day) were also evaluated^21)^.

### 6) Statistical analysis

The Shapiro–Wilk test was used to assess data for normality before and 1 year after SG, and appropriate tests were chosen depending on the results. The Wilcoxon signed-rank test was used to compare changes in blood biochemistry test results, skeletal muscle mass, body fat, muscle strength, and IPAQ scores before and 1 year after SG. Spearman’s rank correlation coefficient was used to investigate how changes in skeletal muscle density (changes in HU of the psoas major muscle [ΔPM-CT] and of the multifidus muscle [ΔMM-CT]) were associated with changes in muscle strength (ΔAMS and ΔRMS) and changes in IPAQ scores (ΔST and ΔTPA), with the change (Δ) calculated as the value before SG subtracted from the value 1 year after SG. EZR (R version 3.4.1; Saitama Medical Center, Jichi Medical University, Saitama, Japan) was used to perform statistical analysis, and p <0.05 was regarded as significant.

## Results

**Fig. 1.**
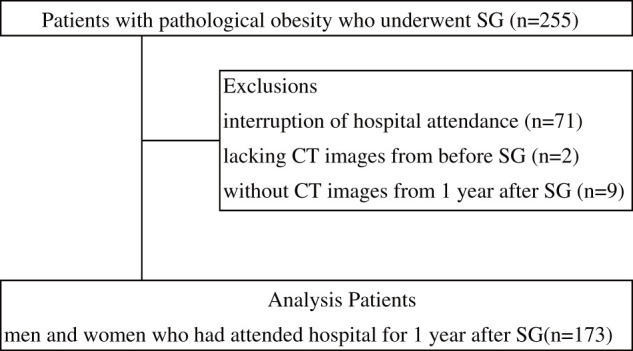
Flowchart of the study population. SG, sleeve gastrectomy; n, number; CT, computed tomography

Figure 1 shows the patient selection process. After excluding 71 patients who stopped attending appointments, 2 lacking CT images from before SG, and 9 without CT images from 1 year after SG, 173 patients were included in the analysis. Patients’ characteristics are shown in Table 1. Their mean age was 46 ± 11 years. Sixty-two men (36%) and 111 women (64%) had comorbidities, including hypertension (52%), dyslipidemia (55%), diabetes mellitus (63%), fatty liver (88%), sleep apnea syndrome (63%), and mental illness (34%).

**Table 1.** Participants’ characteristics

Characteristic	Total (n = 173)
Age (years), mean ± standard deviation	46 ± 11
Sex, male/female	62 (36%)/111 (64%)
Comorbidities	
Hypertension	90 (52%)
Dyslipidemia	96 (55%)
Diabetes mellitus	109 (63%)
Fatty liver	152 (88%)
Sleep apnea syndrome	109 (63%)
Mental illness	59 (34%)

Results are reported as n (%) unless otherwise specified.

n, number

Tables 2 and 3 show the changes in various parameters, including body measurements, blood biochemistry test results, body fat, skeletal muscle mass, skeletal muscle quality, muscle strength measurements, and IPAQ scores from before SG to 1 year after SG. Table 4 shows the associations of changes in skeletal muscle density (ΔPM-CT and ΔMM-CT) with changes in muscle strength (ΔAMS and ΔRMS) and changes in IPAQ scores (ΔST and ΔTPA).

**Table 2.** Changes in various parameters, including body measurements, blood biochemistry test results, and body fat, from before SG to 1 year after SG

	Before SG (n = 173)	1 year after SG (n = 173)	Difference	p Value
Body measurements				
Height (cm)	159.7 (154.9–167.3)			
Weight (kg)	107.4 (93.7–126.5)	83.6 (72.3–97)	−37.7	<0.001
BMI (kg/m^2^)	40.6 (36.6–47.2)	32.4 (28.8–36.1)	−12.9	<0.001
Blood biochemistry tests				
HbA1c (%)	6.2 (5.8–7.1)	5.5 (5.2–5.8)	−1.5	<0.001
FPG (mg/dL)	115 (99–138)	92 (87–100)	−47.8	<0.001
AST (U/L)	30 (21–46)	18 (16–23)	−28.8	<0.001
ALT (U/L)	41 (23–71)	17 (12–23)	−54.4	<0.001
γ-GPT (U/L)	42 (27–74)	20 (14–33)	−34.9	<0.001
HDL-C (mg/dL)	48 (41–56)	60 (50–69)	+12.3	<0.001
LDL-C (mg/dL)	130 (109–149)	121 (99–139)	−26.9	<0.001
TG (mg/dL)	142 (103–200)	87 (66–120)	−61.6	<0.001
HOMA-IR	7.4 (4.7–11.4)	2 (1.4–3.3)	−9.2	<0.001
Body fat				
VFA (cm^2^)	199 (164–251)	105 (76–145)	−109	<0.001
SFA (cm^2^)	484 (388–627)	342 (222–438)	−222.1	<0.001

Data are median (25th–75th percentile) values unless otherwise stated. p Values are from the Wilcoxon signed-rank test.

SG, sleeve gastrectomy; n, number; BMI, body mass index; HbA1c, hemoglobin A1c; FPG, fasting plasma glucose; AST, aspartate transaminase; ALT, alanine transaminase; γ-GPT, gamma-glutamyl transferase; HDL-C, high-density lipoprotein-cholesterol; LDL-C, low-density lipoprotein-cholesterol; TG, serum triglycerides; HOMA-IR, homeostasis model assessment of insulin resistance; VFA, visceral fat area; SFA, subcutaneous fat area

**Table 3.** Changes in various parameters, skeletal muscle mass, skeletal muscle density, muscle strength measurements, and IPAQ scores from before SG to 1 year after SG

	Before SG (n = 173)	1 year after SG (n = 173)	Difference	p Value
Skeletal muscle mass				
PMI (cm^2^/m^2^)	4.52 (3.39–5.65)	3.96 (3.04–4.95)	−0.83	<0.001
Skeletal muscle density				
PM-CT (HU)	39.7 (35.5–42.9)	42.2 (37.5–45.8)	+4	<0.001
MM-CT (HU)	26.1 (16.9–34.7)	35.3 (27.4–41.4)	10.6	<0.001
Muscle strength measurement				
AMS (kgf)	29.9 (25.1–39.3)	29.5 (23.6–40.1)	−1.4	<0.001
RMS (kgf/kg)	0.29 (0.23–0.36)	0.37 (0.3–0.45)	+0.12	<0.001
IPAQ				
ST (hours/day)	14 (12–20.5)	12 (10–19)	−1.3	<0.001
TPA (MET-hours/week)	8.3 (3.3–15.6)	11.6 (5–23.1)	+8.3	<0.001

Data are median (25th–75th percentile) values unless otherwise stated. p Values are from the Wilcoxon signed-rank test.

IPAQ, International Physical Activity Questionnaire; SG, sleeve gastrectomy; n, number; PMI, psoas muscle index; PM-CT, HU in the psoas major muscle; MM-CT, HU in the multifidus muscle; HU, Hounsfield Unit; AMS, absolute muscle strength; RMS, relative muscle strength; ST, sitting time; TPA, total physical activity

**Table 4.** Correlations of the amount of change in muscle strength and changes in IPAQ scores with skeletal muscle density of the psoas major and multifidus muscles

	ΔPM-CT (HU)	p Value	ΔMM-CT (HU)	p Value
	r Value		r Value	
Muscle strength measurement				
ΔAMS (kgf)	−0.08	0.24	−0.08	0.28
ΔRMS (kgf/kg)	0.22	<0.001	0.32	<0.001
IPAQ				
ΔST (hours/day)	−0.11	0.14	−0.16	<0.05
ΔTPA (MET-hours/week)	0.18	<0.05	0.31	<0.001

P value from Spearman's rank-correlation coefficient.

IPAQ, International Physical Activity Questionnaire; PM-CT, HU in the psoas major muscle; MM-CT, HU in the multifidus muscle; HU, Hounsfield Unit; AMS, absolute muscle strength; RMS, relative muscle strength; ST, sitting time; TPA, total physical activity; MET, metabolic equivalent of task

Comparisons of body measurements, blood biochemistry test results, and body fat measurements before and 1 year after SG showed that weight, BMI, HbA1c, FPG, AST, ALT, γ-GPT, LDL-C, TG, HOMA-IR, VFA, and SFA values decreased (p <0.001), whereas HDL-C values increased (p <0.001). Comparisons of skeletal muscle mass, skeletal muscle density, muscle strength, and IPAQ scores before and 1 year after SG showed that the PMI, AMS, and ST values decreased (p <0.001), whereas muscle density (psoas major and multifidus muscle CT values) and TPA values increased (p <0.001) (Tables 2 and 3). Notably, the results showed a significant improvement in the CT values of the multifidus muscle 1 year after SG (Table 3).

On correlation analysis of skeletal muscle density, ΔPM-CT was significantly positively correlated with ΔRMS (r = 0.22, p <0.001) and ΔTPA (r = 0.18, p <0.05). In addition, ΔMM-CT was significantly positively correlated with ΔRMS (r = 0.32, p <0.001) and ΔTPA (r = 0.31,p <0.001), and it was negatively correlated with ΔST (r = −0.16, p <0.05) (Table 4). Notably, the results showed a significant improvement, namely, that the CT values of the multifidus muscle were highly correlated with physical activity and RMS compared with those of the psoas major muscle.

## Discussion

In addition to supporting long-term weight loss, SG has been shown to improve glycemic metabolism and other obesity-related health problems^1,2)^. SG is the most commonly performed procedure worldwide and has been covered by health insurance in Japan since 2014^22)^. In the present study, the associations of skeletal muscle density, which is an indicator of skeletal muscle quality, with muscle strength and physical activity were examined. A decrease in body weight and an improvement in glycolipid metabolism, as well as a decrease in skeletal muscle mass and a decrease in AMS, in the year following SG were observed, whereas there was a shortening of the ST, an increase in RMS, and an increase in TPA (Tables 2 and 3). These results are consistent with those of previous studies^23)^.

In contrast, the study by Kai et al. assessed changes in the PMI and CT values of the psoas major muscle before and 1 year after SG and found a decrease in the PMI 1 year after SG; no changes were observed in the CT values of the psoas major muscle. Physical activity and muscle strength were not evaluated^7)^. In this study, the CT values of the psoas major and multifidus muscles significantly improved 1 year post-SG, likely due to both fat loss and enhanced muscle strength and physical activity during the 1st year post-SG. These improvements in the CT values potentially reflect the overall effect of these factors. Therefore, a comprehensive evaluation, including physical activity and muscle strength that reflects skeletal muscle mass and the quality of skeletal muscle, rather than a single evaluation of body composition alone, is required.

The biological changes observed in this study 1 year after SG are thought to be due to the improvements in insulin sensitivity and glycemic control associated with weight loss. The expected benefits of SG include improvements in obesity-related health problems such as type 2 diabetes mellitus and dyslipidemia. Improvements in glucose and lipid metabolism and the decrease in visceral fat, as well as the increase in RMS due to weight loss, may have led to improvements in exercise tolerance and TPA. Maintaining high levels of physical activity should lead to further improvements in QOL, including physical activity and physical function. The consensus statement of the International Association for the Study of Obesity recommends “at least one 60–90 min of moderate-to-high physical activity per day” to prevent weight gain^24)^. According to a previous study, although ST decreased and physical activity levels increased after SG, patients were unable to achieve the recommended levels of physical activity^25)^. Similarly, the results of the present study showed that, although physical activity levels increased, patients did not achieve the recommended moderate physical activity (23 MET-hours/week) of 60 minutes or more as recommended in the guidelines. Increasing physical activity helps prevent muscle wasting and the accumulation of fat in skeletal muscle^17–19)^.

The key feature of the present study is that it focused not only on quantitative changes in skeletal muscle but also on qualitative changes. Since CT values are valid indicators of skeletal muscle quality due to their reproducibility and objectivity^9,11)^, the higher the CT value, the lower the fat infiltration and the higher the skeletal muscle density, and therefore the higher the muscle quality^9,11)^. One year after SG, there was not only a decrease in PMI but also an improvement in the density of skeletal muscle, i.e., an improvement in quality, as shown by the CT values of the psoas major muscle and the multifidus muscle (Table 3). Furthermore, the results of correlation analysis showed that improvements in skeletal muscle quality, as indicated by the changes in the CT values of target muscles, were associated with increases in RMS and TPA (Table 4). Therefore, the results of the present study suggest that improvements in skeletal muscle quality may be related to improvements in muscle strength and TPA after SG. These results are consistent with previous results showing that increased fat infiltration of skeletal muscle was associated with muscle weakness and reduced physical activity^11,15–19)^, and that, following SG, AMS, as measured by grip strength, either decreased or did not change, but RMS increased at 6–12 months. In a study by Kim et al., weight loss was accompanied by a decrease in skeletal muscle mass and a decrease in AMS, whereas RMS increased^26)^. This study provides novel insights, namely, that the CT values of the multifidus muscle were highly correlated with physical activity and RMS compared with those of the psoas major muscle. These results suggest that skeletal muscle changes vary from muscle to muscle. Although small, significant correlations were observed for psoas major and multifidus muscle CT values. Although these correlations were statistically significant, the correlation coefficients (r-values) were modest. This suggests that the relationships between muscle quality and physical function and strength are complex aspects influenced by multiple factors. Nevertheless, even such relatively low correlations may indicate the importance of muscle quality in changes to body composition after SG. The literature shows that the multifidus muscle is composed of Type I fibers (slow muscle), with obese individuals having fewer Type I fibers and more fatty infiltration. These individuals are also more prone to fatty degeneration progression and qualitative changes, while the CT values reflect less fat accumulation in the psoas major muscle than in the multifidus muscle^27–29)^. In this context, the CT values of the multifidus muscle suggest that weight loss after an SG may indicate reduced visceral and subcutaneous adipose tissues, improved skeletal muscle density, increased physical activity, and improved RMS.

This study has several limitations. First, it was conducted at a single institution with a limited number of patients. Second, this was a pilot observational study. The timings of initial outpatient presentation and regular follow-ups were inconsistent, and patients did not undergo a CT evaluation phase. Third, physical activity levels were evaluated via a paper questionnaire, without an objective assessment. Fourth, the results of the present study suggest that improvements in skeletal muscle quality were a significant predictor of, or may be related to, improvements in muscle strength and TPA after SG in our univariate analysis, but not in the multivariate analysis. Possible reasons for this included the morphologic differences between men and women, or age. A prospective, observational study with more patients and future in-depth investigations, including long-term observational studies, studies with a control group, and studies that use activity monitors, will be necessary to address these limitations.

Future studies will need to examine pre- and postexercise interventions, measure lower extremity muscle strength, and use a physical activity meter to elucidate any potential causal relationships.

## Conclusions

The improvement in skeletal muscle quality that occurs after SG may lead to an increase in RMS and an improvement in TPA.
